# Development and application of new composite grouting material for sealing groundwater inflow and reinforcing wall rock in deep mine

**DOI:** 10.1038/s41598-018-23995-y

**Published:** 2018-04-04

**Authors:** Zhang Jinpeng, Liu Limin, Zhang Futao, Cao Junzhi

**Affiliations:** 1 College of Mining and Safety Engineering, Shandong University of Science and Technology, Qingdao, 266590 China; 2 Shandong ark New Material Co., Ltd, Tai’an, Shandong, Taian 271026 China; 3 Ark Mines Ltd. Tai’an, Shandong, Taian 271026 China

## Abstract

With cement, bentonite, water glass, J85 accelerator, retarder and water as raw materials, a new composite grouting material used to seal groundwater inflow and reinforce wall rock in deep fractured rock mass was developed in this paper. Based on the reaction mechanism of raw material, the pumpable time, stone rate, initial setting time, plastic strength and unconfined compressive strength of multi-group proportion grouts were tested by orthogonal experiment. Then, the optimum proportion of composite grouting material was selected and applied to the grouting engineering for sealing groundwater inflow and reinforcing wall rock in mine shaft lining. The results show the mixing proportion of the maximum pumpable time, maximum stone rate and minimum initial setting time of grout are A_K4_B_K1_C_K4_D_K2_, A_K3_B_K1_C_K1_D_K4_ and A_K3_B_K3_C_K4_D_K1_, respectively. The mixing proportion of the maximum plastic strength and unconfined compressive strength of grouts concretion bodies are A_K1_B_K1_C_K1_D_K3_ and A_K1_B_K1_C_K1_D_K1_, respectively. Balanced the above 5 indicators overall and determined the optimum proportion of grouts: bentonite-cement ratio of 1.0, water-solid ratio of 3.5, accelerator content of 2.9% and retarder content of 1.45%. This new composite grouting material had good effect on the grouting engineering for sealing groundwater inflow and reinforcing wall rock in deep fractured rock mass.

## Introduction

The grouting technique has been widely applied in the field of geotechnical engineering, including controlling groundwater inrush and reinforcing wall rock in underground mine, subsea tunnel, foundation pit, slope, dam body and so on. The grouts is injected into the fractured water-bearing rock stratum or incompact water-bearing sand layer. The grout gel can fill the fracture or the joint plane of the rock stratum and sand layer, thus effectively sealing groundwater inrush and reinforcing the rock stratum and sand layer. Grouting technology mainly includes. grouting process, grouting materials and grouting equipment^1^. The grouting material is the main material to consolidate the rock stratum and sand layer. So, it is the key to reinforce wall rock and seal groundwater inflow.

Currently, the research for grouting materials mainly includes the strength mechanism^2,3^, the workability^4–6^, and the admixture^7,8^. Based on the working performance of ordinary grouting material, some special grouting materials were put forward according to different geological conditions. Basing on plastic viscosity and yield stress model, Shimada *et al*.^9^ developed the grouting material used for the floor reinforcement in underground mines. Song *et al*.^10^ developed a series of high performance & multi-functional agent (Hi-FA) grout- ing materials in an underwater condition. Yang *et al*.^11^ made an organic-inorganic hybrid chemical grouting material with “flexible and stretchable” approach. Zhang *et al*.^12^ developed a new cement-based materials, which was made up of cement and SJP admixture. Zhang *et al*.^13^ prepared a new double liquid grouting material- superfine sulfoaluminate cement grouting material. Wei *et al*.^14^ invented a new grouting material based on sulfoaluminate cement (SAC) and investigated its properties under pressure circulation.

The above grouting materials are special materials for special geological conditions. Therefore, they are not universal and extensive. In general, the above grouting material is mainly divided into chemical material and cement-based materials. Chemical grouting material has the characteristics of high price and partial toxicity, so it is not suitable for engineering application. Ordinary cement-based materials is cheap, but it has the characteristics of low stone rate, low strength and long setting time. So, they can not be widely used in grouting reinforcement engineering.

In addition, with the depth of mine and underground engineering increasing, the ground stress increases gradually and the geological conditions become more and more complex, leading to the geological disasters such as collapse and water inrush are more and more frequent. Therefore, the requirements for the strength, setting time, stone rate and durability of grouting material are higher. However, the above grouting materials can not fully meet the requirements for sealing groundwater inflow and reinforcing wall rock in deep fractured rock mass^15–17^. So, it is necessary to develop a new and more effective grouting material for sealing groundwater inflow and reinforcing wall rock in deep fractured rock mass.

In order to solve the problem of difficult grouting for sealing groundwater inflow and reinforcing wall rock in deep fractured rock mass, in this paper, a new composite grouting material was developed and applied to the grouting engineering in deep mine. The raw materials of this new composite grouting material mainly include cement, bentonite, water glass, J85 accelerant, retarder and water. The new composite grouting material has the characteristics of high stone rate, high strength of grouts concretion bodies, long pumpable time and short setting time. It is an ideal grouting material for sealing groundwater inflow and reinforcing wall rock in deep fractured rock mass.

## Raw Material and Reaction Mechanism

### Raw material

In this experiment, the Ordinary Portland cement (P.O. 42.5) produced by Shimen Cement Company, China was in use and its strength characteristics was confirmed to GB/T 17671-1999. The bentonite were produced by Shandong ark New Material Co., Ltd. The water glass solution with the modulus of 2.4–3.4 and the mass concentration of 30–45Be was produced by Chengdu Hongrui Chemical Co., Ltd. The mixed solution of J85 and water glass with the volume ratio of 1.1 was selected as the accelerator. NH_4_H_2_PO_4_ was used as the retarder. The water was obtained from tap water.

### Grouts reaction mechanism

1. Cement and bentonite. Bentonite is highly dispersed in cement grouting. It can bridge each other into the network structures that make a large amount of free water into irreducible water, thereby forming a non-Newtonian liquid-type thixotropic gel. Bentonite can improve the water retention and stability of the grouts. Therefore, bentonite is beneficial to the grouts for sealing groundwater inflow.
2. Cement and J85 accelerator. The J85 accelerator can inhibit the retarding effect of gypsum on cement grouting and generate indissolvable salt and aluminite to accelerate grouts condensation. The reaction is as follows.
  1 $${{\rm{Na}}}_{2}{{\rm{CO}}}_{3}+{\rm{CaO}}+{{\rm{H}}}_{2}{\rm{O}}\to {{\rm{CaCO}}}_{3}+2{\rm{NaOH}}$$
  2 $${{\rm{Na}}}_{2}{{\rm{CO}}}_{3}+{{\rm{CaSO}}}_{4}\to {{\rm{CaCO}}}_{3}+{{\rm{Na}}}_{2}{{\rm{SO}}}_{4}$$
3. Water glass and bentonite. Water glass solution reacts with alkaline earth metal in bentonite to produce alkali metal hydrous silicate and SiO2 gel. The reaction is as follows.
  3 $${{\rm{Na}}}_{2}{\rm{O}}\cdot {{\rm{nSiO}}}_{2}+{{\rm{CaCL}}}_{2}+{{\rm{xH}}}_{2}{\rm{O}}\to 2{\rm{NaCL}}+{{\rm{CaSiO}}}_{3}\cdot {{\rm{xH}}}_{2}{\rm{O}}+({\rm{n}}-1){{\rm{SiO}}}_{2}$$
  4 $${{\rm{Na}}}_{2}{\rm{O}}\cdot {{\rm{nSiO}}}_{2}+{{\rm{MgCL}}}_{2}+{{\rm{xH}}}_{2}{\rm{O}}\to 2{\rm{NaCL}}+{{\rm{MgSiO}}}_{3}\cdot {{\rm{xH}}}_{2}{\rm{O}}+({\rm{n}}-1){{\rm{SiO}}}_{2}$$
  5 $${{\rm{Na}}}_{2}{\rm{O}}\cdot {{\rm{nSiO}}}_{2}+{{\rm{BaCL}}}_{2}+{{\rm{xH}}}_{2}{\rm{O}}\to 2{\rm{NaCL}}+{{\rm{BaSiO}}}_{3}\cdot {{\rm{xH}}}_{2}{\rm{O}}+({\rm{n}}-1){{\rm{SiO}}}_{2}$$
  SiO_2_ gel and hydrous silicate can increase the cohesive force between bentonite particles and increase the strength of the complex formed by the grouts concretion bodies and the rock mass. So, it is beneficial to the grouts for reinforcing wall rock.
4. Cement and water glass. The water glass reacts with Ca(OH)_2_ in cement grouting to produce the gel (calcium silicate hydrate) with a certain strength. The reaction is as follows.
  6 $${\rm{Ca}}{({\rm{OH}})}_{2}+{{\rm{Na}}}_{2}{\rm{O}}\cdot {{\rm{nSiO}}}_{2}+{{\rm{mH}}}_{2}{\rm{O}}\to {\rm{CaO}}+{{\rm{nSiO}}}_{2}\cdot {{\rm{mH}}}_{2}{\rm{O}}+{\rm{NaOH}}$$
  The hydration reaction of 3CaO · SiO_2_ and 2CaO · SiO_2_ in cement grouting generates Ca(OH)_2_. The solubility of Ca(OH)_2_ is low, which reduces the hydration reaction rate of 3CaO · SiO_2_ and 2CaO · SiO_2_. However, water glass can increase the hydration reaction rate of 3CaO · SiO2 and 2CaO · SiO2, shorten the initial setting time of grouts and improve the early strength of grouts concretion bodies.
5. According to the hypothesis of inhibition nucleation, the retarder can block the nucleation of Ca(OH)_2_ in the liquid and decrease the hydration reaction rate of 3CaO · SiO2. So as to play a retarding role.

## Orthogonal Experiment of Grouts

### Experimental design

Orthogonal experimental design is a design method to study the multi-factor and multi-level. It is based on the orthogonality to select some representative points from the comprehensive experiment. The orthogonal experimental design is the main method of fractional factorial design. So, orthogonal experiment was used to investigate the multiple target parameters of the mix proportion of raw materials in this paper.

The grouts concretion bodies can improve load-carrying capacity of fractured rock mass and recover the continuity of rock mass. According to the stress characteristics of the fractured rock mass, the concretion bodies should possess high plastic strength and compressive strength. In order to improve the grouting effect on fractured rock mass, the grouts should possess long pumpable time and short initial setting time. There are many factors influencing the performance of grouts. Among these, bentonite-cement ratio, water-solid ratio, accelerator content and retarder content are important ones.

According to the application experience and related data^18–20^, preliminarily determined that the water-cement ratio of the grouts is 0.8–1.2, the water-solid ratio (the ratio of cement and bentonite to grout) is 3.5–5.0, the bentonite-cement ratio is 1–1.6, the accelerator content is cement and bentonite (solid) of 2.0–2.9%, the retarder content is cement and bentonite (solid) of 1.0–1.9%. The effect of different bentonite-cement ratio, water-solid ratio, accelerator content and retarder content on the pumpable time, stone rate, initial setting time, plastic strength and unconfined compressive strength of grouts were investigated by orthogonal experiment. The orthogonal experiment scheme was designed with four factors at four levels, and the test parameters are as follows.

According to the orthogonal design table L_16_(4^4^), the test scheme is shown in Table 1, in which the factors A,B,C and D were bentonite-cement ratio, water-solid ratio, accelerator content and retarder content, respectively. The four levels of A were 1.0, 1.2, 1.4, 1.6, four level of B were 3.5, 4.0, 4.5, 5.0, four level of C were 2.0%, 2.3%, 2.6%, 2.9% and four levels of D were 1.0%, 1.3%, 1.6%, 1.9%, respectively.

**Table 1:** Orthogonal experimental scheme L_16_(4^4^).

Group	A. bentonite-cement ratio	B. water-solid ratio	C. accelerator content (%)	D. retarder content (%)
1	1.0	3.5	2.0	1.0
2	1.0	4.0	2.3	1.3
3	1.0	4.5	2.6	1.6
4	1.0	5.0	2.9	1.9
5	1.2	3.5	2.3	1.6
6	1.2	4.0	2.0	1.9
7	1.2	4.5	2.9	1.0
8	1.2	5.0	2.6	1.3
9	1.4	3.5	2.6	1.9
10	1.4	4.0	2.9	1.6
11	1.4	4.5	2.0	1.3
12	1.4	5.0	2.3	1.0
13	1.6	3.5	2.9	1.3
14	1.6	4.0	2.6	1.0
15	1.6	4.5	2.3	1.9
16	1.6	5.0	2.0	1.6

## Experimental Method

The pumpable time, stone rate, initial setting time, plastic strength and unconfined compressive strength of grouts were tested respectively. In order to reduce the deviations, each set of experiments was repeated 3 times. The average of the 3 sets of data was used as the test result. The experimental method is as follows.

1. Pumpable time
  Pour the well stirred grouts into the conic-shaped mold and pick up the mold. Measured the maximum diameter of the grouts flowing freely on a glass plane. The period of time from 0 to the state that the maximum diameter of grouts is less than 14 cm is the pumpable time. As shown in Fig. 1(a).

**Figure 1:**
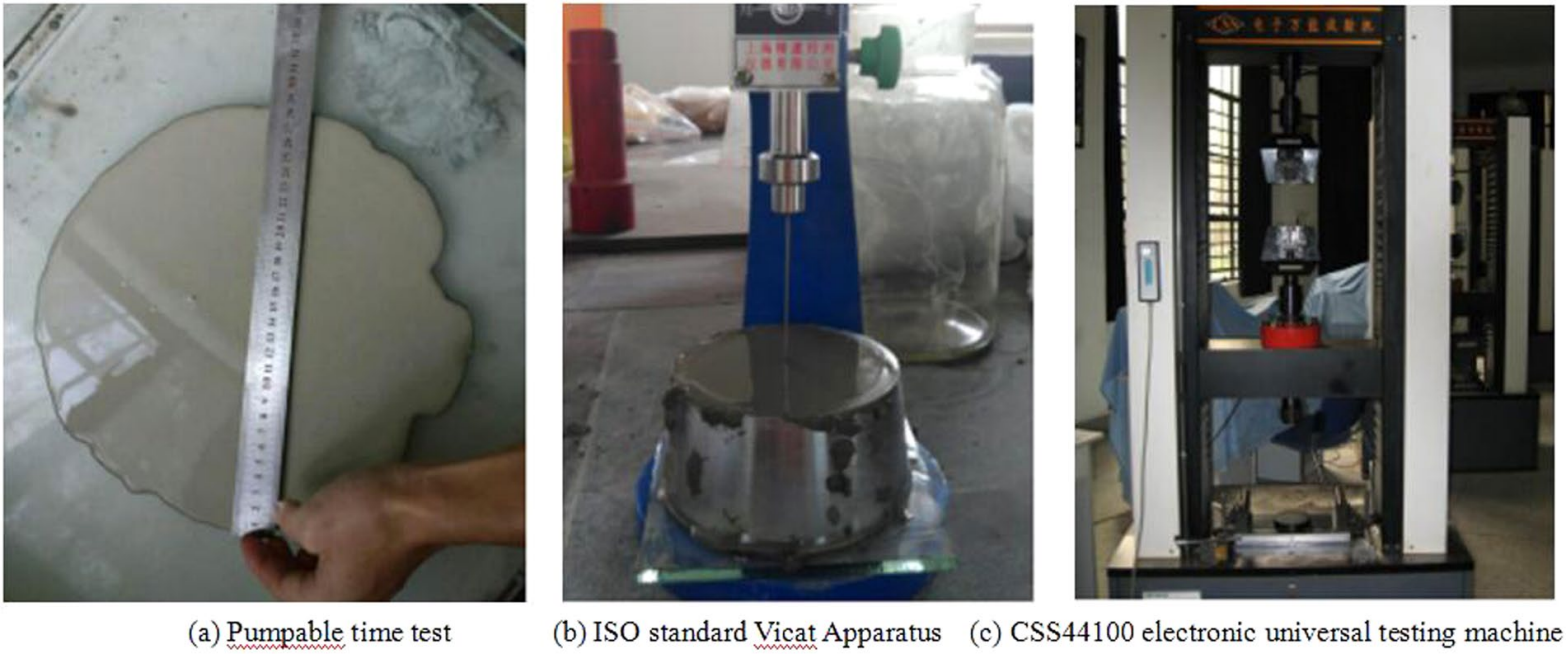
Experimental device and facility.
2. Stone rate
  Pour the well stirred grouts into the testing mold of 100 mm × 100 mm × 100 mm. After grouts solidification, measure the average height of specimen by vernier caliper. The volume ratio of grouts concretion bodies to original grouts is the stone rate.
3. Initial setting time
  The initial setting time of grouts was tested by using “ISO standard Vicat Apparatus”. Figure 1(b) is ISO standard Vicat Apparatus.
4. Plastic strength and unconfined compressive strength

Pour the well stirred grouts into the testing mold of 4 × 4 × 8 cm3. After grouts solidification and demoulding, put the specimen in the water to maintain. The plastic strength and unconfined compressive strength of the grouts concretion bodies at 3, 7, 14 and 28 days were measured by 300-kN CSS44100 electronic universal testing machine, respectively. Figure 1(c) is CSS44100 electronic universal testing machine.

## Results and Discussion

### Range analysis method

*R* is the range value for the pumpable time, stone rate, initial setting time, plastic strength and unconfined compressive strength of grouts. *R* represents the influence degree of the factors on the target parameters.

7 $$R={K}_{imax}-{K}_{imix}$$

Where, *K*_*i*_ is the average value of experimental value for each factor in the i-th level.

### Variance analysis method

*K*_*h*_ is the sum of experimental value for each factor in the h-th level, h = 1,2,3. T is the sum of the experimental value for each test schemes. C is correction. *S*_*j*_ is the square of deviance of each factor (j is A,B,C,D). S_T_ is total square of deviance. S_e_ is square of deviance of error.

8 $$C={T}^{2}/n$$

9 $${S}_{j}=({{K}_{1}}^{2}+{{K}_{2}}^{2}+{{K}_{3}}^{2})/3-C.$$

10 $${S}_{T}=\sum _{j=1}^{9}{{y}_{j}}^{2}-C$$

11 $${S}_{{\rm{e}}}={S}_{{\rm{T}}}-{S}_{{\rm{A}}}-{S}_{{\rm{B}}}-{S}_{{\rm{C}}}-{S}_{D}.$$

The ratio of the square of deviance of each factor to the square of deviance of error is defined as *F. F* represents the influence degree of each factor on the target parameters.

12 $$F=\frac{{S}_{j}/{f}_{j}}{{S}_{{\rm{e}}}/{f}_{e}}$$

where, *S*_*j*_ and *f*_*j*_ is square of deviance and degree of freedom of each factor. *S*_*e*_ and *f*_*e*_ is square of deviance and degree of freedom of error.

### Pumpable time, stone rate and initial setting time of grouts

**Figure 2:**
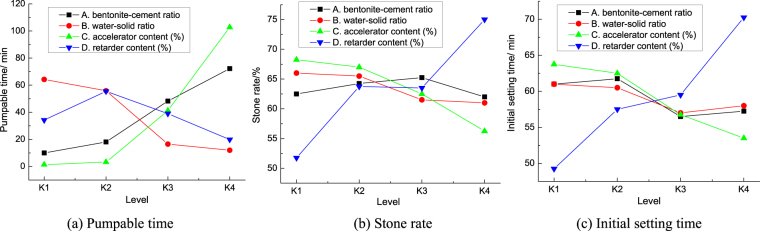
Average pumpable time, stone rate and initial setting time of grouts at different level.

**Figure 3:**
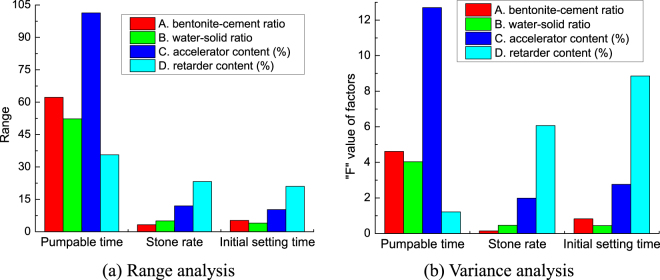
Range analysis and variance analysis of the pumpable time, stone rate and initial setting time of the grouts.

According to the test data in Table 2, the average pumpable time, stone rate and initial setting time of grouts at different level is in Fig. 2. Then, the pumpable time, stone rate and initial setting time of the grouts are analyzed by range analysis and variance analysis. The range and variance (“F” range) of the pumpable time, stone rate and initial setting time of the grouts is in Fig. 3. Figure 3 show that the influence law of each factor on the target parameters in range analysis is basically the same as that in variance analysis.

**Table 2:** The data table of orthogonal experiment results.

Group	A	B	C	D	Pumpable time/min	Stone rate/%	Initial setting time/min	Plastic strength /KPa				Unconfined compressive strength /KPa			
								f3	f7	f14	f28	f3	f7	f14	f28
1	1	3.5	2.0	1.0	3	58	58	452	625	1172	1187	368.1	487.8	519.2	526.1
2	1	4.0	2.3	1.3	1	62	60	385	532	1082	1116	332.5	436.4	458.1	466.8
3	1	4.5	2.6	1.6	12	62	58	354	475	1024	1036	304.5	389.6	405.8	412.4
4	1	5.0	2.9	1.9	24	68	68	332	430	977	997	283.6	347.5	358.2	361.6
5	1.2	3.5	2.3	1.6	4	74	70	372	461	928	964	318.2	407.6	432.1	440.3
6	1.2	4.0	2.0	1.9	0.5	85	80	348	411	901	916	293.5	380.5	403.7	411.1
7	1.2	4.5	2.9	1.0	48	37	40	326	405	838	855	278.5	353.7	373.1	379.1
8	1.2	5.0	2.6	1.3	20	61	57	305	365	786	809	265.5	328.5	343.6	348.5
9	1.4	3.5	2.6	1.9	50	72	63	315	386	811	821	269.1	354.4	371.7	381.2
10	1.4	4.0	2.9	1.6	139	60	53	294	337	758	790	252.4	325.8	347.7	360
11	1.4	4.5	2.0	1.3	1	72	60	275	306	659	678	239.2	296.4	317.2	325.3
12	1.4	5.0	2.3	1.0	3	57	50	255	283	584	613	227.7	268.9	290.8	300.7
13	1.6	3.5	2.9	1.3	200	60	53	275	329	730	751	223.2	316.2	323.5	334.5
14	1.6	4.0	2.6	1.0	83	55	49	257	301	692	725	207.1	295.7	300.9	308.2
15	1.6	4.5	2.3	1.9	5	75	70	238	279	668	687	191.7	277.1	279.7	285.3
16	1.6	5.0	2.0	1.6	1	58	57	221	262	643	671	174.5	257.6	261.3	266.1

Figures 2 and 3 shows that:

In the range analysis and variance analysis of pumpable time, the influence degree of each factor on the pumpable time of grouts is in the order: accelerator content > bentonite-cement ratio > water-solid ratio > retarder content. Among them, the accelerator content has the greatest influence on the pumpable time of grouts. The influence degree of bentonite-cement ratio on pumpable time is slightly larger than that of water-solid ratio. The influence degree of retarder content is relatively small. With the increase of the accelerator content and bentonite-cement ratio, the pumpable time of grouts both increases gradually. With the increase of the water-solid ratio, the pumpable time of grouts decreases gradually. It shows that the pumpable time of grouting material can be adjusted by the accelerator content, water-solid ratio and bentonite-cement ratio. In addition, when the retarder content is 1.3%, the grouts have the longest pumpable time. So, the mixing proportion of the maximum pumpable time of grouts is A_K4_B_K1_C_K4_D_K2_.

In the range analysis and variance analysis of the stone rate, the influence degree of each factor on the stone rate of grouts is in the order: retarder content > accelerator content > water-solid ratio > bentonite-cement ratio. Among them, the retarder content has the greatest influence on the stone rate of grouts. The bentonite-cement ratio and water-solid ratio have little influence on the stone rate of grouts. Retarders can reduce the hydration reaction rate of cement, so that the distribution of hydration products in grouts is more uniform, which is beneficial to the full hydration of the cement particles. So, with the increase of the retarder content, the stone rate of grouts increases gradually. The accelerator content can increase the hydration reaction rate of cement, which is not beneficial to the full hydration of the cement particles. So,with the increase of the accelerator content, the stone rate of grouts decreases gradually. It shows that the stone rate of grouts can be adjusted by the retarder content and accelerator content. The mixing proportion of the maximum stone rate of grouts is A_K3_B_K1_C_K1_D_K4_.

In the range analysis and variance analysis of the initial setting time, the influence degree of each factor on the initial setting time of grouts is in the order: retarder content > accelerator content > bentonite-cement ratio > water- solid ratio. Among them, the retarder content has the greatest influence on the initial setting time of grouts; the bentonite-cement ratio and water-solid ratio have little influence on the initial setting time of grouts. With the increase of the retarder content, the initial setting time of grouts increases gradually. The reason is that retarder can block the nucleation of Ca(OH)_2_ in the liquid and decrease the hydration reaction rate of 3CaO · SiO_2_. With the increase of the accelerator content, the initial setting time of grouts decreases gradually. The reason is that the accelerator can accelerate the hydration reaction rate of 3CaO · SiO_2_ and 2CaO · SiO_2_, thus shortening the initial setting time of grouts. It shows that the initial setting time of grouting material can be adjusted by the retarder content and accelerator content. The mixing proportion of the minimum initial setting time of grouts is A_K3_B_K3_C_K4_D_K1_.

### Plastic strength of grouts concretion bodies

**Figure 4:**
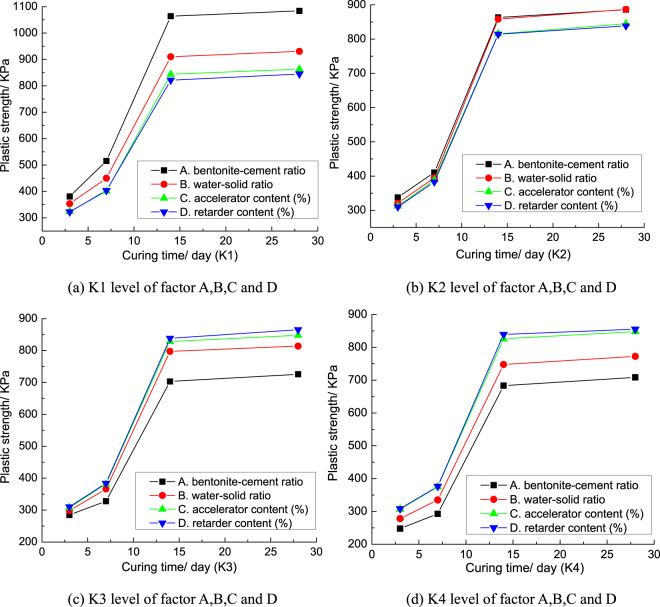
Average plastic strength of grouts concretion bodies at different curing times.

According to the test data in Table 2, the average plastic strength of grouts concretion bodies at different curing times is in Fig. 4.

From Fig. 4, it can be understood that with the increase of curing time, the plastic strength of grouts concretion bodies increased gradually. The increasing degree of the plastic strength of grouts concretion bodies was not large from 3 days to 7 days. But the plastic strength of grouts concretion bodies increased rapidly during 7 days to 14 days. The plastic strength of grouts concretion bodies at 14 days was about twice that of grouts concretion bodies at 7 days, which showed that the plastic strength of grouts concretion bodies was good at 14 days. There was no obvious change in the plastic strength of grouts concretion bodies from 14 days to 28 days, which showed that the grouts concretion bodies had a strong stability.

**Figure 5:**
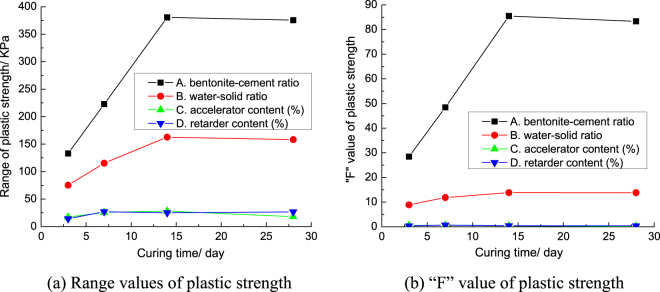
The broken line drawing for the range analysis and variance analysis of plastic strength.

In order to compare and analyze the influence of the four factors on the plastic strength of grouts concretion bodies, the range and variance (“F” value) of the plastic strength for each factor are drawn into the broken line, as shown in Fig. 5. Figure 5 show that the influence law of each factor on the plastic strength of grouts concretion bodies in range analysis is basically the same as that in variance analysis.

Figure 5 shows that the bentonite-cement ratio and water-solid ratio have the outstanding influence on the plastic strength of grouts concretion bodies. But the retarder content and accelerator content have little influence on the plastic strength of grouts concretion bodies. The influence degree of bentonite-cement ratio and water-solid ratio on the plastic strength of grouts concretion bodies increased gradually during 3 days to 14 days. After 14 days, the influence degrees of bentonite-cement ratio and water-solid ratio on the plastic strength of grouts concretion bodies were basically stable. Bentonite can improve the adaptive deformation capacity of grouts concretion bodies. From Fig. 4, we can know that with the increasing of bentonite-cement ratio and water-solid ratio, the plastic strength of grouts concretion bodies decreases gradually. So, the plastic strength of grouts concretion bodies can be adjusted by bentonite-cement ratio and water-solid ratio. As the curing time goes on, the influence degrees of accelerator content and retarder content on the plastic strength of grouts concretion bodies increased at first and then stabilized. The influence degree of accelerator content and retarder content on the plastic strength of grouts concretion bodies are both the most outstanding at 7 days. In order to maintain the long-term stability of grouts concretion bodies, the grouts concretion bodies must have high plastic strength after 28 days. So, the level of each factor with the highest plastic strength of grouts concretion bodies at 28 days is chosen as the best level. The mixing proportion of the maximum plastic strength of grouts concretion bodies is A_K1_B_K1_C_K1_D_K3_.

### Unconfined compressive strength of grouts concretion bodies

**Figure 6:**
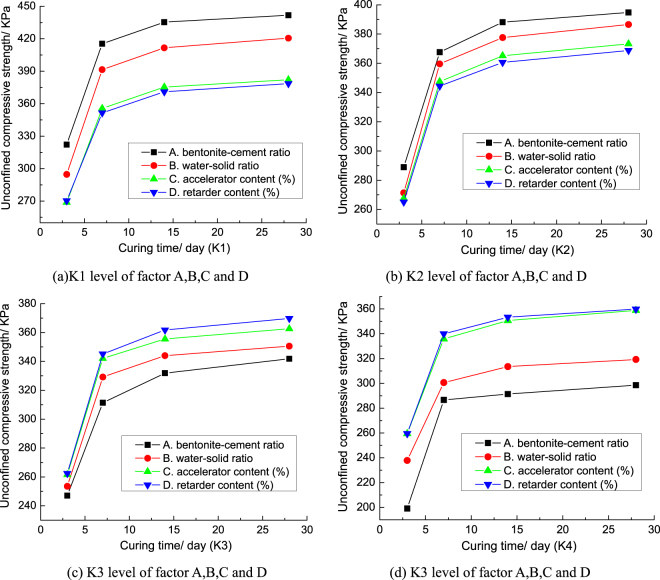
Average unconfined compressive strength of grouts concretion bodies at different curing times.

According to the test data in Table 2, the average unconfined compressive strength of grouts concretion bodies at different curing times is in Fig. 6.

Figure 6 shows that the unconfined compressive strength of grouts concretion bodies increased rapidly from 3 days to 7 days. The unconfined compressive strength of grouts concretion bodies increased slowly from 7 days to 14 days. After 14 days, the unconfined compressive strength of grouts concretion bodies increased more slowly and reached maximum at 28 days. It shows that the grouting material has the characteristics of high early strength, which confirms that the accelerator can improve the early strength of grouts concretion bodies. The main reason is: the solidification of grouts in the early stage was due to the effect of the accelerator. As time goes by, the strength of grouts concretion bodies was further increased due to the effect of the cement and bentonite. So, the bentonite-cement ratio has the outstanding influence on the unconfined compressive strength of grouts. It also showed that the unconfined compressive strength of the grouts concretion bodies reached its maximum value soon after the solidification of grouts. And the grouts concretion bodies can maintain the long-term stability of the maximum unconfined compressive strength.

**Figure 7:**
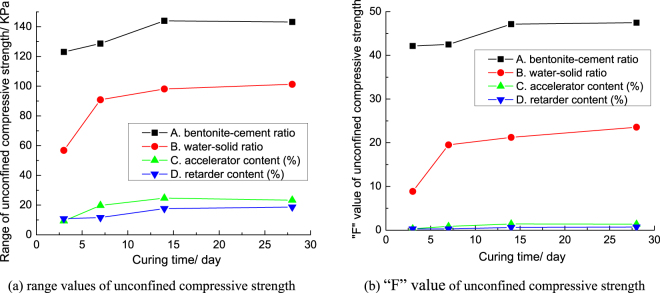
The broken line drawing for the range analysis and variance analysis of unconfined compressive strength.

In order to compare and analyze the influence degree of the four factors on the unconfined compressive strength of grouts concretion bodies, the range and variance (“F” value) of the unconfined compressive strength for each factor are drawn into the broken line, as shown in Fig. 7. Figure 7 show that the influence law of each factor on the unconfined compressive strength of grouts concretion bodies in range analysis is basically the same as that in variance analysis.

Figure 7 shows that the bentonite-cement ratio and water-solid ratio have the greatest influence on the unconfined compressive strength of grouts concretion bodies; the retarder content and accelerator content have little influence on the unconfined compressive strength of grouts concretion bodies. The influence order of each factor on the unconfined compressive strength of grouts is similar to that of each factor on the plastic strength of grouts concretion bodies. The influence degree of bentonite-cement ratio and water-solid ratio on the unconfined compressive strength of grouts concretion bodies were both increased gradually during 3–14 days. After 14 days, the influence degrees of bentonite-cement ratio and water-solid ratio on the unconfined compressive strength of grouts concretion bodies were basically stable. The influence degree of the retarder content and accelerator content on the unconfined compressive strength of grouts concretion bodies varies little with the curing time. The hydration reaction of bentonite is not obvious, so the ability of bentonite increasing the strength of grouts concretion bodies is far less cement. The more water in grouts, the lower the density of the solid particles. So, with the increasing of bentonite-cement ratio and water-solid ratio, the unconfined compressive strength of grouts concretion bodies both decreases gradually. The unconfined compressive strength of grouts concretion bodies can be adjusted by bentonite-cement ratio and water-solid ratio. The level of each factor with the highest unconfined compressive strength of grouts concretion bodies at 28 days is chosen as the best level. So, the mixing proportion of the maximum unconfined compressive strength of grouts concretion bodies is A_K1_B_K1_C_K1_D_K1_.

### Comprehensive analysis of orthogonal experiment

The analysis of orthogonal experiment show that the mixing proportions of the maximum pumpable time, the maximum stone rate and the minimum initial setting time of grouts is A_K4_B_K1_C_K4_D_K2_, A_K3_B_K1_C_K1_D_K4_, A_K3_B_K3_C_K4_D_K1_, respectively. The mixing proportions of the maximum plastic strength and the maximum unconfined compressive strength of grouts concretion bodies is A_K1_B_K1_C_K1_D_K3_ and A_K1_B_K1_C_K1_D_K1_, respectively.

According to the sequence of influence degree of each factor and best level combination, the comprehensive balance of the optimum ratio in the 5 characteristics of grouts was carried out. Then, selected the best level of the greatest influence factor on the target parameters as the best level of the factor. Finally, the optimum ratio of the grouts which is most favorable to the 5 characteristics is put forward. The analysis shows that the best levels of factor A, factor B and factor C are K1, K1 and K4, respectively. The greatest influence factors of stone rate and initial setting time are both D, and the corresponding best levels of stone rate and initial setting time are K4 and K1, respectively. Therefore, the middle level K2 or K3 should be taken. The optimum mixing proportion of the grouts is A_K1_B_K1_C_K4_D_K2,3_. Thus, the proportion of the new composite grouting material is bentonite-cement ratio of 1.0, water-solid ratio of 3.5, accelerator content of 2.9% and retarder content of 1.45%.

## Engineering Application

The buried depth of Jinyan Coal Mine is 1061 m. The thickness of topsoil and the diameter of shaft are 566.38 m and 4.6 m, respectively. The freezing method was used for the shaft construction. The thickness of the reinforced concrete in the shaft lining is 0.76 m. The groundwater inflow occurred in the process of mine production. the maximum water inflow was 15m^3^/h. The reason of the water inflow is that the fractured rock mass in the shaft lining connected the Quaternary aquifer. The water inflow increased gradually with the increase of fracture width, resulting in the large range destruction of shaft lining.

Due to the large buried depth and complicated geological structure of the mine, the ordinary cement- based grouting material can not effectively reinforce wall rock and seal groundwater inflow. So, the new new composite grouting material in this paper was used in the shaft lining for sealing groundwater inflow and reinforcing wall rock. The results show that when the grout amount was 40t, the shaft lining stopped the water inflow.

The new composite grouting material has obvious advantages for sealing groundwater inflow and reinforcing wall rock. Firstly, the grouts have good groutability. When the ordinary cement- based grouting was used in the shaft lining for sealing groundwater inflow and reinforcing wall rock, the grouting pressure was about 5–6 MPa and the maximum pressure even reached 9 MPa. However, when the new composite grouting material was used in the shaft lining, the grouting pressure is about 2-3 MPa and the grouting pressure is stable. It shows that the new composite grouting material has good fluidity and permeability. Secondly, the new composite grouting material has long pumpable time, short initial setting time and high grouts concretion bodies strength. Thirdly, the new composite grouting material has good controllability and stability. The shaft lining has no water inflow for a long time.

**Figure 8:**
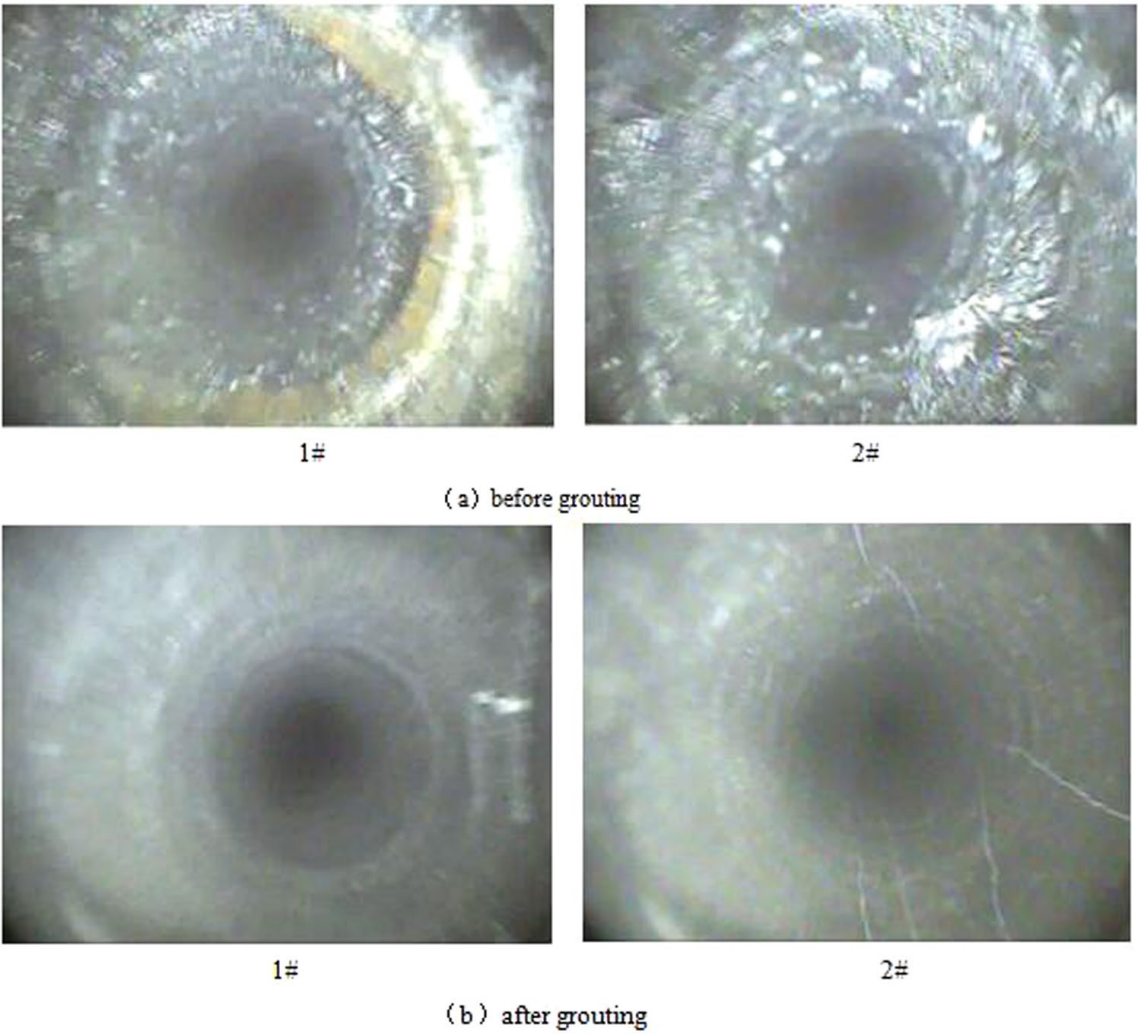
The peeping result of the surrounding rock of the shaft lining before and after grouting.

The new composite grouting material effectively blocked up the cracks and reinforced the fractured rock mass of the shaft lining. So as to achieve the purpose of sealing groundwater inflow and reinforcing wall rock. In order to analyze the influence of the new composite grouting material on the internal structure of surrounding rock, the borehole televiewer was used to detect the internal structure of surrounding rock before and after grouting. Figure 8 is the detecting figure of the internal structure of surrounding rock in the shaft lining before and after grouting. Figure 8 shows that before grouting, there were many fractures and joints in the surrounding rock of the shaft lining; After grouting, the new composite grouting material effectively filled the fractures and joint surfaces of the surrounding rock, thus effectively reinforcing the surrounding rock of the shaft lining.

## Materials and Methods

The new composite grouting material with high stone rate, high strength, long pumpable time and short initial setting time was developed by orthogonal experiment. Then, it is applied to the grouting engineering for sealing groundwater inflow and reinforcing wall rock in deep mine. The following conclusions could be drawn:

Cement, bentonite, J85 accelerator and water glass react in water to form indissolvable salt, aluminite, alkali metal hydrous silicate and SiO_2_ gel. They is beneficial to the grouts for sealing groundwater inflow and reinforcing wall rock. The analysis of orthogonal experiment show that the mixing proportion of the maximum pumpable time, maximum stone rate, minimum initial setting time, maximum plastic strength and maximum unconfined compressive strength of grouts is A_K4_B_K1_C_K4_D_K2_, A_K3_B_K1_C_K1_D_K4_, A_K3_B_K3_C_K4_D_K1_, A_K1_B_K1_C_K1_D_K3_ and A_K1_B_K1_C_K1_D_K1_, respectively. According to the sequence of influence degree on each factor and best level combination, determine the optimum combination of grouts. bentonite-cement ratio of 1.0, water-solid ratio of 3.5, accelerator content of 2.9% and retarder content of 1.45%. That is the proportion of the new composite grouting material for sealing groundwater inflow and reinforcing wall rock. This new composite grouting material had better effect than ordinary cement-based grouting material for sealing groundwater inflow and reinforcing wall rock in deep fractured rock mass. The new composite grouting material effectively filled the fractures and joint planes of the surrounding rock in the shaft lining^21–23^.

### Data availability

The data in manuscript is availability.
